# Supplementary material for: Interplay of mechanics and chemistry governs wear of diamond-like carbon coatings interacting with ZDDP-additivated lubricants
Source: Nat Commun. 2021 Jul 27;12:4550. doi: 10.1038/s41467-021-24766-6 (PMC8316475; doi:10.1038/s41467-021-24766-6)
Supplement: Supplementary file 1 — Supplementary Information [file 41467_2021_24766_MOESM1_ESM.pdf]

Supplementary Information

# Interplay of mechanics and chemistry governs wear of diamond-like carbon coatings with ZDDP-additivated lubricants

Valentin R. Salinas Ruiz[1,2,3†], Takuya Kuwahara[4†], Jules Galipaud[1,2], Karine Masenelli-Varlot[2], Mohamed Ben Hassine[1], Christophe Héau[3], Melissa Stoll[4], Leonhard Mayrhofer[4], Gianpietro Moras[4], Jean Michel Martin[1], Michael Moseler[4,5,6,7*], and Maria-Isabel de Barros Bouchet[1*]

[1]*University of Lyon, Ecole Centrale de Lyon, Laboratory of Tribology and System Dynamics, CNRS UMR5513, 69134 Ecully, France*

[2]*University of Lyon, INSA- Lyon, UCBL, MATEIS UMR CNRS 5510, Villeurbanne, France*

[3]*HEF/IREIS, Avenue Benoît Fourneyron, Andrézieux-Bouthéon, France*

[4]*Fraunhofer Institute for Mechanics of Materials IWM, MicroTribology Center µTC, Wöhlerstraße 11, 79108 Freiburg, Germany*

[5]*Cluster of Excellence livMatS, Freiburg Center for Interactive Materials and Bioinspired Technologies, University of Freiburg, Georges-Köhler-Allee 105, 79110 Freiburg, Germany*

[6]*Institute of Physics, University of Freiburg, Hermann-Herder-Straße 3, 79104 Freiburg, Germany*

[7]*Freiburg Materials Research Center, University of Freiburg, Stefan-Meier-Straße 21, 79104 Freiburg, Germany*

*Corresponding authors. E-mail: michael.moseler@iwm.fraunhofer.de (M.M.)

E-mail: maria-isabel.de-barros@ec-lyon.fr (M.I.D.B.B.)

**Supplementary Note 1. AFM topographies and calculations of root-mean-square heights and slopes**

20μm × 20μm AFM topographies were measured with a resolution of 512×512 pixels. The discretization size $\Delta l$ of our AFM data is about 39 nm – slightly above the diameter of the AFM tip ($d_{\mathrm{tip}} \approx 20$ nm). Note, that discretization smaller than the tip size is prone to mapping errors. In contrast, increasing the grid spacing would lead to the loss of nanoscale characteristics of the surface and as a result numerical contact mechanics calculations would give smaller local contact pressures. Since our interest is in nanoscale contact pressures, the spacing should be as small as possible without biased by AFM errors. Moreover, the RMS slope $h'_{\mathrm{rms}}$ is a central quantity in the Persson's theory[2]. While $h_{\mathrm{rms}}$ is dominated by power-law scaling behaviour of a power spectral density at the largest wavelengths, $h'_{\mathrm{rms}}$ depends entirely on the surface structure at a smallest scale. A comparison of the RMS slope $h'_{\mathrm{rms}}$ between a 20μm × 20μm topography with 512×512 pixels and a 5μm × 5μm topography with 256×256 pixels (corresponding to $\Delta l \approx 20$ nm) for a ta-C(51)-coated disc shows that the calculated values are very similar and thus indicates that the original grid size $\Delta l \approx 39$ nm is sufficiently small.

Supplementary Figure 1 shows AFM surface topographies after running-in inside the wear tracks on both the cylinder and disc, which were used in our contact mechanics calculations (Fig. 1e and f). During running-in, large asperities are removed and thus $h_{\mathrm{rms}}$ and $h'_{\mathrm{rms}}$ decrease for the three softer DLCs (a-C:H, a-C, and ta-C(51)). For example, $h'_{\mathrm{rms}}$ values measured outside the wear tracks for a-C:H are about twice as large as those inside the weak tracks after running-in. In contrast, for ta-C(66) and ta-C(78), wear events are observed especially at the edge of the stroke after 2000 sliding cycles. Supplementary Figure 1d and e show scratches on the cylinder and disc due to wear, resulting in an increase in $h_{\mathrm{rms}}$ and $h'_{\mathrm{rms}}$.

A height map $h_{x,y}$ of a surface topography with $N_x \times N_y$ grid points along the $x$ and $y$ axis was obtained using AFM. The root-mean-square height $h_{\mathrm{rms}}$ and slope $h'_{\mathrm{rms}}$ were computed by the following standard definitions:

$$h_{\mathrm{rms}} = \langle |h|^2 \rangle = \sqrt{\frac{1}{N_x N_y} \sum_x \sum_y \left(h_{x,y} - \langle h \rangle\right)^2}, \tag{1}$$

$$h'_{\text{rms}} = \langle|\nabla h|^2\rangle = \sqrt{\frac{1}{(N_x-1)\cdot(N_y-1)}\sum_x\sum_y\left(\left(\frac{\partial h_{x,y}}{\partial x}\right)^2 + \left(\frac{\partial h_{x,y}}{\partial y}\right)^2\right)}. \quad (2)$$

We used a first-order finite-difference approximation for computing the partial derivatives in Supplementary Eq. 2. The computed RMS heights and slopes for the DLC topographies in Supplementary Fig. 1 are tabulated in Supplementary Table 1.

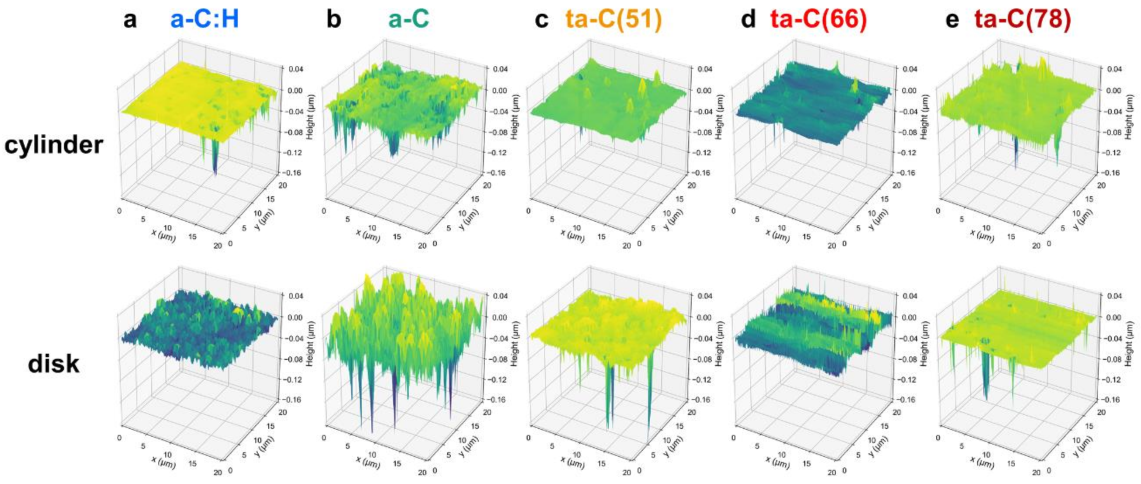


**Supplementary Fig. 1: AFM surface topographies of DLC surfaces coated on the steel cylinder (top) and disc (bottom).** (a) a-C:H, (b) a-C, (c) ta-C(51), (d) ta-C(66) and (e) ta-C(78). For all panels, $\langle h\rangle = 0.0$ µm.

**Supplementary Table 1: RMS heights (nm) and slopes for DLC topographies in Supplementary Fig. 1.**

| | RMS after running-in | | | |
|---|---|---|---|---|
| | height (nm) | | slope (-) | |
| Material | cylinder | disc | cylinder | disc |
| a-C:H | 5.48 | 5.25 | 0.061 | 0.038 |
| a-C | 10.66 | 26.44 | 0.071 | 0.128 |
| ta-C(51) | 4.82 | 10.39 | 0.026 | 0.073 |
| ta-C(66) | 4.22 | 8.87 | 0.051 | 0.084 |
| ta-C(78) | 11.78 | 5.86 | 0.082 | 0.063 |

**Supplementary Note 2. Wear tracks after sliding tests in PAO + ZDDP of ta-C(66) and ta-C(78)**

The wear tracks of the softer DLCs, a-C:H, a-C, and ta-C(51), are barely visible and their wear volumes are negligible in both PAO and PAO+ZDDP (Fig. 1). However, the hardest DLCs, ta-C(66) and ta-C(78), exhibit severe wear and appear very scratched only in PAO+ZDDP. Supplementary Figure 2 shows optical interferometer images of the wear tracks of ta-C(66) and ta-C(78) in PAO+ZDDP. The wear depths of the discs and cylinders is approximately 0.5 and 1.5 µm, respectively. These correspond to about 25% and 75% of the initial film thickness of the DLC coatings ($h_{\mathrm{DLC}} = 2.0$ µm).

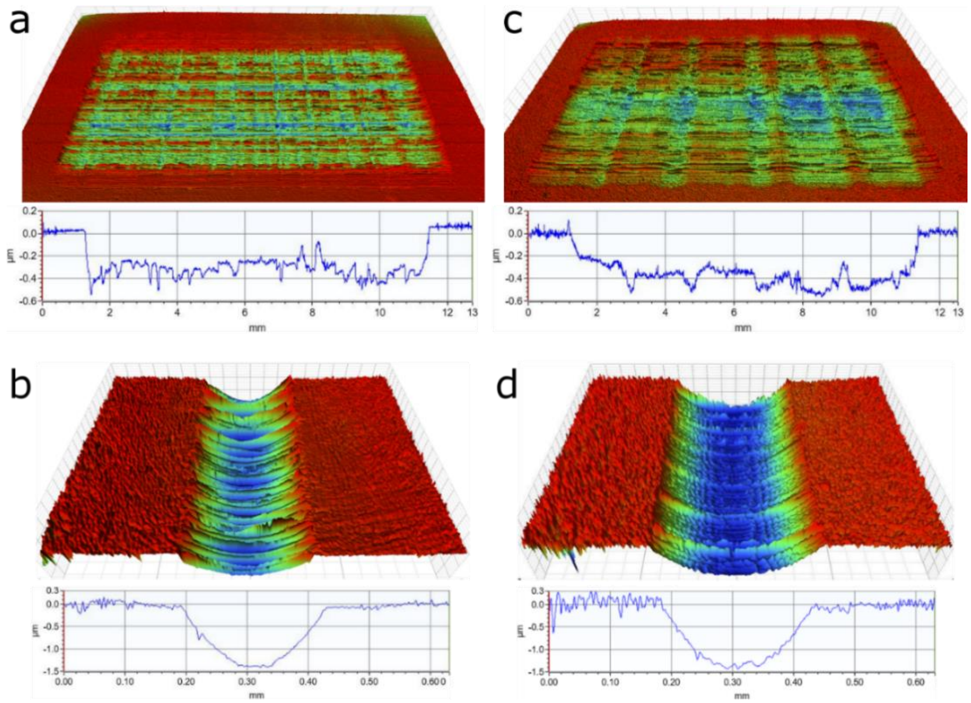


**Supplementary Fig. 2: Optical interferometer images of the wear tracks.** (a) Disc of ta-C(66), (b) cylinder of ta-C(66), (c) disc of ta-C(78), and (d) cylinder of ta-C(78). The images of the cylinders are flattened in order to observe the wear volume.

**Supplementary Note 3. Surface chemistry of DLCs studied by XPS after sliding tests in PAO+ZDDP**

The chemical states of C, O, P and Zn are practically undistinguishable between the five DLCs (see Supplementary Fig. 3). C1*s* XPS spectra show the contribution of three types of chemical bonds:

the first peak at BE = 284.8 eV is attributed to both $sp^2$ and $sp^3$ carbon bonds in DLC as well as to adsorbed aliphatic carbon. The second and third peaks at BE = 286.5 and 288.3eV correspond to C−O and C=O bonds, respectively. O1*s* XPS spectra can be fitted using only two peaks: the first peak at BE = 531.7 eV is attributed to non-bridging oxygen (C=O, oxygen in phosphates and sulphates) and the second peak at BE = 533.0 eV is attributed to C−O bonds. P2*p* spectra are fitted with one doublet with a separation of 0.84 eV and intensity ratio of 0.5. The P2$p_{3/2}$ peak located at BE = 133.4 eV corresponds to phosphates. The obtained P2*p* spectra indicate the absence of poly-phosphates, and thus no contribution from bridging-oxygen is considered. The Zn2$p_{3/2}$ peak at BE = 1022.3 eV corresponds to ZnS or $Zn^{2+}$ in zinc phosphates. No ZnO compound, corresponding to a peak at a lower BE (~1021.2 eV), is detected.

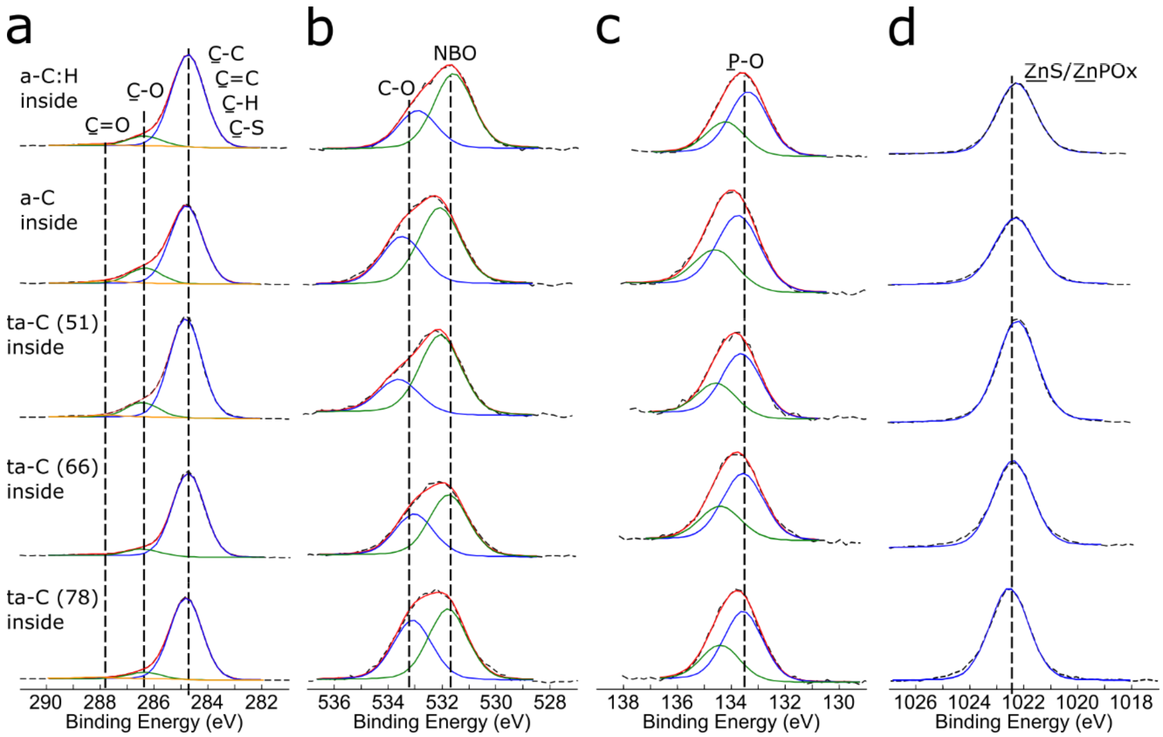


**Supplementary Fig. 3: XPS spectra of DLC surfaces after sliding tests in PAO+ZDDP.** (a) C1*s*, (b) O1*s*, (c) P2*p*, and (d) Zn2$p_{3/2}$ XPS spectra.

**Supplementary Note 4. Analysis on the ZDDP-derived tribofilms formed on DLC surfaces lubricated with PAO+ZDDP**

Supplementary Figure 4 shows representative SEM images of the five DLC surfaces in secondary electrons (SE) detection mode. EDX spectra acquired in different regions of interest allow the identification of chemical species. The spectra are indicated by red or green dashed regions or points. The green spectra are used to identify the ZDDP-derived tribofilm (bright tribo-patches) and the red ones for the DLC surface (gray areas). The quantification results of the recorded spectra are presented in Supplementary Table 2.

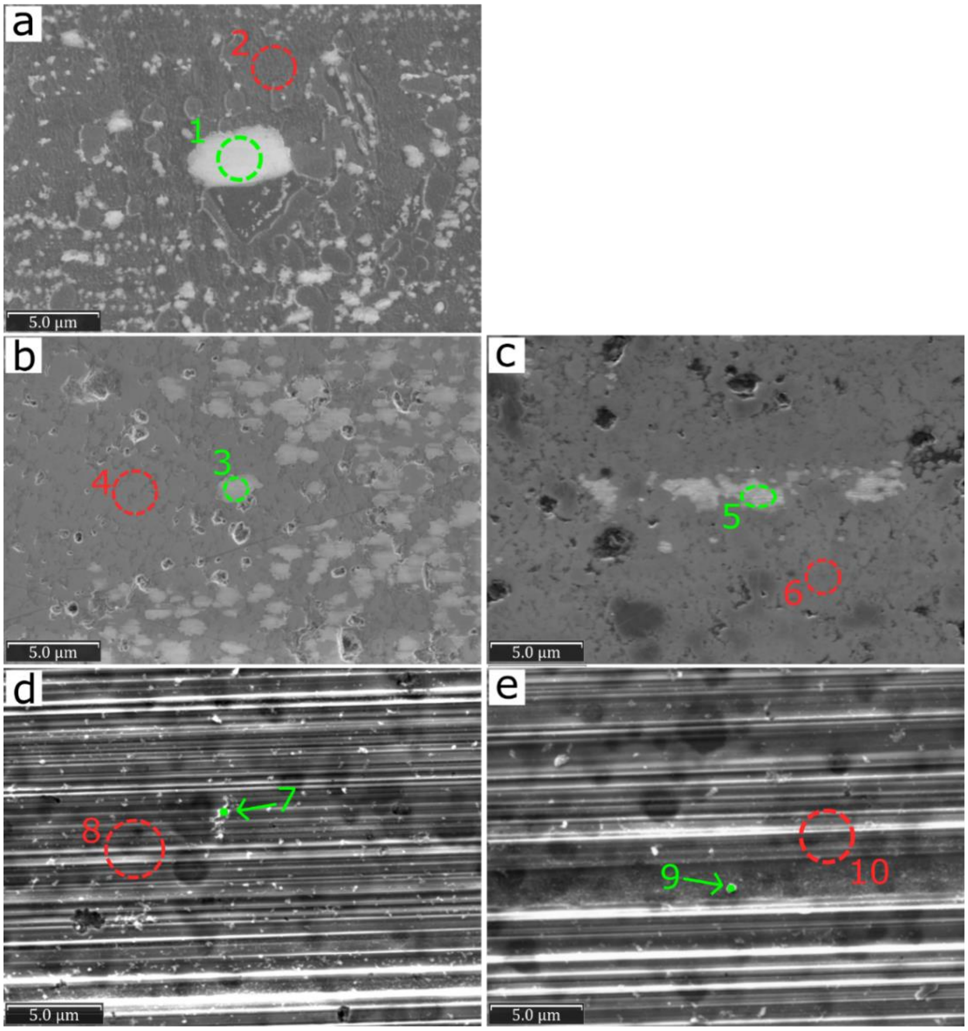


**Supplementary Fig. 4: SEM images of DLC surfaces after sliding tests in PAO+ZDDP in SE detection mode.** Red dashed-circles and dots indicate the regions analyzed by EDX spectra. (a) a-C:H, (b) a-C, (c) ta-C(51), (d) ta-C(66) and (e) ta-C(78).

**Supplementary Table 2: Elemental quantification obtained by EDX analysis of a-C:H and ta-C in the regions shown in Supplementary Fig. 4 after tribological experiments in PAO+ZDDP.**

| | Chemical composition (at.%) | | | | |
|---|---|---|---|---|---|
| Spectrum # | C | O | P | S | Zn |
| Spectrum 01 | 84.3 | 5.0 | 2.8 | 1.9 | 6.0 |
| Spectrum 02 | 99.3 | 0.7 | - | - | - |
| Spectrum 03 | 88.3 | 3.2 | 1.6 | 2.0 | 4.9 |
| Spectrum 04 | 99.8 | 0.2 | - | - | - |
| Spectrum 05 | 93.9 | 1.4 | 0.3 | 1.7 | 2.7 |
| Spectrum 06 | 99.7 | 0.3 | - | - | - |
| Spectrum 07 | 98.8 | 0.2 | 0.3 | 0.6 | 0.1 |
| Spectrum 08 | 99.8 | 0.2 | - | - | - |
| Spectrum 09 | 96.1 | 2.0 | 0.5 | 1.1 | 0.3 |
| Spectrum 10 | 99.9 | 0.1 | - | - | - |

It is also interesting to note that ZDDP-derived tribo-patches form predominantly near the edges of the stroke and much less are found at the middle of the wear track (Supplementary Fig. 5). This indicates that probably due to weak interactions of ZDDP with DLC a boundary lubrication condition is a prerequisite for either tribofilm formation or sulphur doping of the DLC surface. In contrast, on steel, thick tribofilms form even without asperity contacts, i.e. under hydrodynamic lubrication[1].

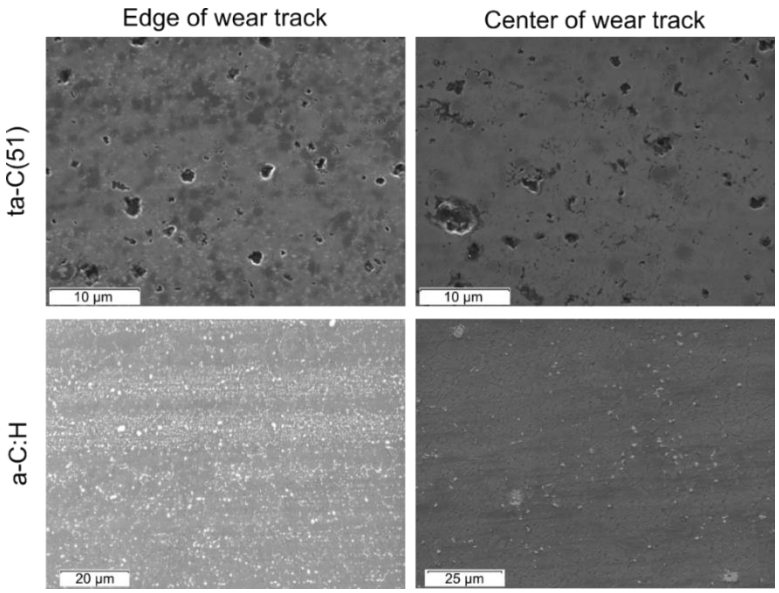


**Supplementary Fig. 5: SEM images in SE detection mode after sliding tests.** The top and bottom panels represent SEM images for ta-C(51) and a-C:H, respectively. SEM images in the left and right column are those measured at the edge and centre of the wear track, respectively.

## Supplementary Note 5. Elemental quantification obtained by EDX analysis of a-C:H and ta-C(66) in the regions shown in Fig. 4 after tribological experiments in PAO+ZDDP.

Supplementary Table 3 shows the quantification results of the spectra recorded in ROI 1−3 for a-C:H and ROI 4−8 for ta-C(66). The ROIs are indicated in Fig. 4.

**Supplementary Table 3: Elemental quantification of the spectra recorded in ROI 1−3 for a-C:H and ROI 4−8 for ta-C(66).**

| Label | ROI | Elemental quantification of the spectra (at.%) | | | | |
|---|---|---|---|---|---|---|
| | | C | O | S | Zn | Pt |
| a-C:H | 1 | 65.7 | 22.0 | 5.1 | 5.0 | 2.2 |
| | 2 | 66.1 | - | - | - | 33.9 |
| | 3 | 100.0 | - | - | - | - |
| ta-C(66) | 4 | 94.7 | 2.8 | 1.2 | 1.3 | - |
| | 5 | 92.0 | 5.4 | 1.7 | 0.9 | - |
| | 6 | 93.7 | 4.6 | 1.1 | 0.6 | - |
| | 7 | 99.5 | 0.5 | - | - | - |
| | 8 | 99.3 | 0.7 | - | - | - |

### Supplementary Note 6. Quasi-static contact-closing/opening simulations of fully-hydrogen-passivated DLC surfaces

Usually, asperities on DLC surfaces are sufficiently passivated with hydrogen atoms or other functional groups[5]. During asperity contacts hydrogen terminations of DLC surfaces via dehydrogenation of alkyl groups in ZDDP can form, which is one of the dominant mechanochemical reactions at higher contact pressures. However, asperity collisions can simultaneously remove passivating species mechanically and produce reactive carbon atoms[11]. Therefore, we consider unpassivated surfaces in the main text of this article. In this note, we examine the reactivity of hydrogen-passivated surfaces. For fully-H-passivated surfaces, C−C bonds across the cutting plane were replaced with hydrogen atoms when introducing the vacuum region. Supplementary Figure 6 shows averaged numbers of S−C bonds $\langle \Delta n_{\mathrm{S-C}} \rangle$ formed between ZDDP sulphur and DLC carbon atoms and sulphur atoms released from ZDDP $\langle n_{\mathrm{Sulphur}} \rangle$ to the DLC after opening the contact as a function of the contact pressure $P_z$ for both a-C:H and a-C. There is a clear difference in the reactivity of ZDDP with DLC surfaces between unpassivated (Fig. 6) and hydrogen-passivated surfaces (Supplementary Fig. 6). The formation of S−C bonds and release of sulphur to DLC surfaces are severely inhibited for both hydrogen-passivated a-C:H and a-C surfaces especially at $P_z \lesssim 10$ GPa. The difference in the chemical structures of DLC surfaces also has a minor role in ZDDP decomposition for hydrogen-passivated surfaces.

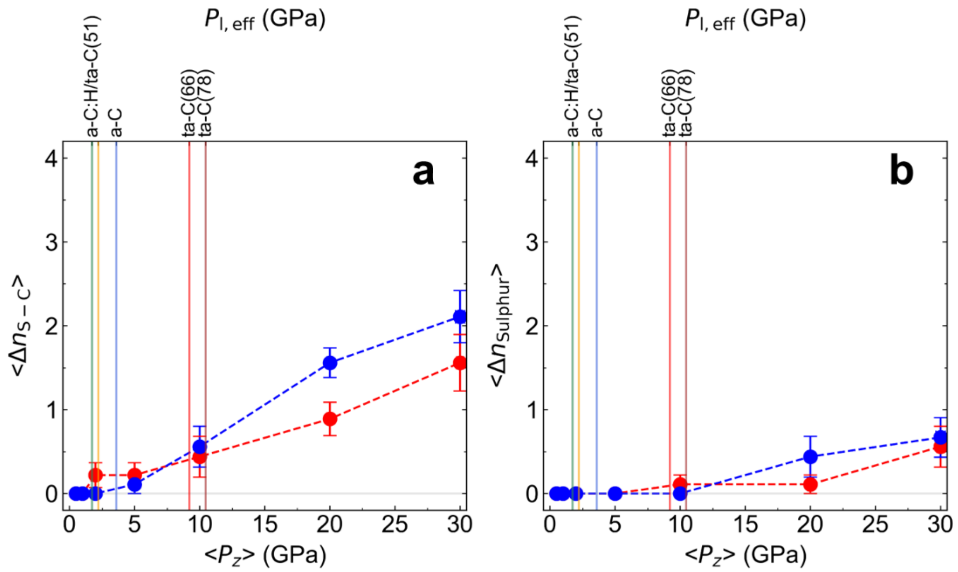


**Supplementary Fig. 6: Quasi-static contact-closing/reopening simulations for hydrogen-passivated a-C:H with $\rho$ = 2.0 g cm$^{-3}$ and $C_H$ = 20 at.% and for hydrogen-passivated a-C with $\rho$ = 2.0 g cm$^{-3}$ in contact with a ZDDP molecule using DFTB3 quantum chemical calculations.** Averaged numbers of (a) S−C bonds $\langle \Delta n_{S-C} \rangle$ formed between ZDDP sulphur and DLC carbon atoms as well as (b) sulphur atoms released from ZDDP $\langle n_{Sulphur} \rangle$ to the DLC after opening the contact as a function of the contact pressure $P_z$ for a-C:H (red) and a-C (blue). The error bars represent standard error of the means. The vertical lines mark the effective local contact pressures $P_{l,eff}$ for the five experimental coatings (reported in Fig. 1f).

## Supplementary Note 7. Quasi-static contact-closing/opening simulations of *sp*$^3$-rich ta-C surfaces

Hard ta-Cs contain more *sp*$^3$ carbon atoms in the bulk compared with soft a-Cs. However, the *sp*$^3$ content in the top surface region is completely different from that in the bulk, and the surface is covered with an *sp*$^2$-rich a-C layer. According to a previous molecular dynamics study by Kunze et al.[11], sliding ta-C and even diamond produces a surface a-C region with a low *sp*$^3$ content of about 10%, evidenced by experiments in the same article. Therefore, we chose a-C systems with low *sp*$^3$ contents (less than 10%) in our DFTB simulations. Larger *sp*$^3$ contents near surfaces would not be representative of a tribological DLC surface. Nevertheless, it would be interesting to study the effect of the *sp*$^3$ content in the top surface region on ZDDP' reactions. We thus performed

quasi-static contact-closing/opening simulations for 9 ta-C systems with higher *sp*$^3$ contents (ranging from 48 to 67%) and a density of 3.0 g cm$^{-3}$. Supplementary Figure 7 shows that the average number of S−C bonds and sulphur released onto the surface and their pressure dependence are similar to those for a-C:H and a-C. This indicates that an increase in the *sp*$^3$ content on the DLC surface has a minor influence on the chemical reactivity of ZDDP (especially S-C bonds formation and sulphur release). Thus, we can conclude that the kinetics of ZDDP's reactions on unpassivated DLC surfaces is predominantly determined by the local contact pressure.

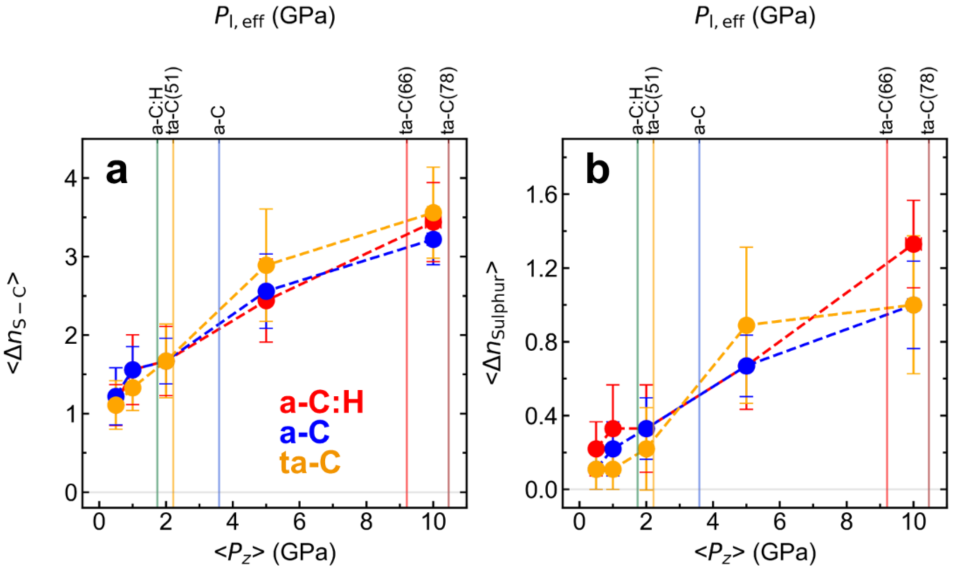


**Supplementary Fig. 7: Quasi-static contact-closing/reopening simulations for *sp*$^3$-rich ta-C with $\rho$ = 3.0 g cm$^{-3}$ (orange) in contact with a ZDDP molecule using DFTB3 quantum chemical calculations.** Averaged numbers of (a) S−C bonds $\langle \Delta n_{S-C} \rangle$ formed between ZDDP sulphur and DLC carbon atoms as well as (b) sulphur atoms released from ZDDP $\langle n_{\mathrm{Sulphur}} \rangle$ to the DLC after opening the contact as a function of the contact pressure $P_z$. The plots for a-C:H (red) and a-C (blue) are the same as in Fig. 6. The error bars represent standard error of the means. The vertical lines mark the effective local contact pressures $P_{\mathrm{l,eff}}$ for the five experimental coatings (reported in Fig. 1f).

## Supplementary Note 8. ZDDP-derived fragments and their contact pressure dependence

As we discussed in the main text, ZDDP decomposition is not influenced by the structural and chemical differences between a-C:H and a-C, but by the local contact pressure $P_\mathrm{l}$ (Supplementary Fig. 8). At low $P_\mathrm{l}$ ($\lesssim$ 1 GPa), most ZDDP molecules undergo fragmentation into two chemical species. An increase in $P_\mathrm{l}$ accelerates decomposition, and sulphur and hydrogen of alkyl groups are predominantly released onto DLC surfaces. The detachment of alkyl groups is a rate-limiting step. For a-C:H, $P_z = 10$ GPa, the surface is functionalized with ZDDP-derived species. The accumulation of ZDDP-derived species could lead to the growth of anti-wear tribofilms. In contrast, ta-C undergoes cold-welding and subsequent mechanical mixing of the sub-surface, resulting in the sulphur doping in the matrix as well as the formation of polyynic chains after surface separation.

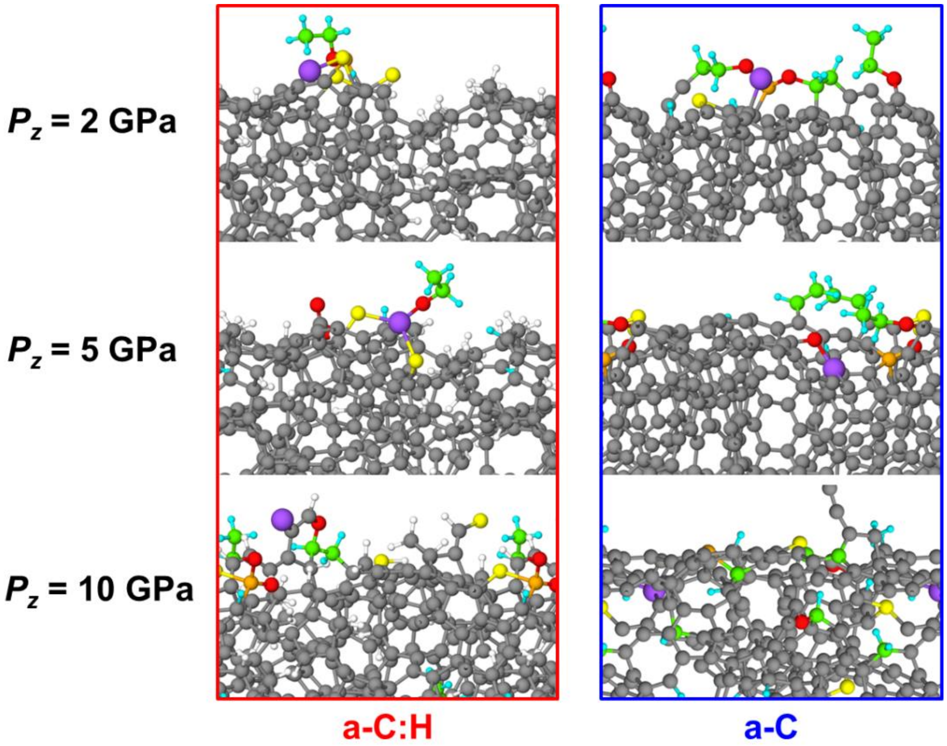


**Supplementary Fig. 8: Representative pictures of DLC surfaces functionalized with ZDDP-derived atoms/fragments.** Atomic configurations for a-C:H (left) and a-C (right) at three different contact pressures ($P_z$ = 2, 5, and 10 GPa) after opening the contacts.

**Supplementary Note 9. Estimation of the friction regimes**

In our reciprocating friction tests, the sliding speed $v$ is not constant. At the edges of the stroke (where there is a change of direction), $v$ falls to zero, whereas $v$ reaches its maximum value ($v \approx 0.157$ ms$^{-1}$) in the center of the stroke. This means that at the edges of the stroke, much less lubricant is entrained in the contact zone between two contacting bodies, resulting in a transition from mixed/hydrodynamic lubrication (ML/HL) to boundary lubrication (BL). To estimate the friction regime of our sliding tests, we performed elastohydrodynamic lubrication (EHL) film thickness calculations using a Moes' equation for line contacts[12] and calculated Tallian's lambda values[13]. The central EHL film thickness $H_{\mathrm{EHL}}$ is calculated by

$$H_{\mathrm{EHL}} = \left[\left(H_{\mathrm{RI}}^{7/3} + H_{\mathrm{EI}}^{7/3}\right)^{3s/7} + \left(H_{\mathrm{RP}}^{-7/2} + H_{\mathrm{EP}}^{-7/2}\right)^{-2s/7}\right]^{s^{-1}}, \qquad (3)$$

where s is an auxiliary variable defined as $s = \frac{1}{5}\left(7 + 8\exp(-2\,H_{\mathrm{EI}}/H_{\mathrm{RI}})\right)$, and $H_{\mathrm{RI}}$, $H_{\mathrm{EI}}$, $H_{\mathrm{RP}}$, and $H_{\mathrm{EP}}$ are also defined as $H_{\mathrm{RI}} = 3M^{-1}$, $H_{\mathrm{EI}} = 2.621M^{-1/5}$, $H_{\mathrm{RP}} = 1.287L^{2/3}$, $H_{\mathrm{EP}} = 1.311M^{-1/8}L^{3/4}$, respectively. The dimensionless lubricant number $L$ and load number $M$ are defined as $\alpha E'\left(\frac{\eta_0 v}{E'R}\right)^{1/4}$ and $\frac{F}{E'R}\left(\frac{E'R}{\eta_0 v}\right)^{1/2}$, where $E'$ is the reduced Young's modulus ($\frac{2}{E'} = \frac{1-\upsilon_1^2}{E_1} + \frac{1-\upsilon_2^2}{E_2}$), $v$ is the sliding speed, $\eta_0$ is the lubricant viscosity, $\alpha$ is the viscosity pressure coefficient, $R$ is the radius of the cylinder, and $F$ is the normal load per unit length. The Tallian's lambda values $\lambda$ is defined as $\frac{H_{\mathrm{EHL}}}{h_{\mathrm{rms}}}$, where $h_{\mathrm{rms}}$ is the composite root-mean-square roughness of the cylinder and disc (defined as $h_{\mathrm{rms}} = \sqrt{\left(h_{\mathrm{rms,cylinder}}\right)^2 + \left(h_{\mathrm{rms,disc}}\right)^2}$). Here, $h_{\mathrm{rms,cylinder}}$ and $h_{\mathrm{rms,disc}}$ are taken from Table 1 for all DLC coatings. Supplementary Figure 9 shows a visual representation of the friction regimes in our reciprocating sliding tests for a-C:H and ta-C(51). BL ($\lambda < 1$) and ML ($1 \leq \lambda < 3$) apply at the edges and middle of the stroke, respectively. In our a-C:H system, about 18% of the stroke are under BL, where tribochemical reactions of ZDDP with DLC surfaces occur. For ta-C(51), the BL regime accounts for 32% of the stroke.

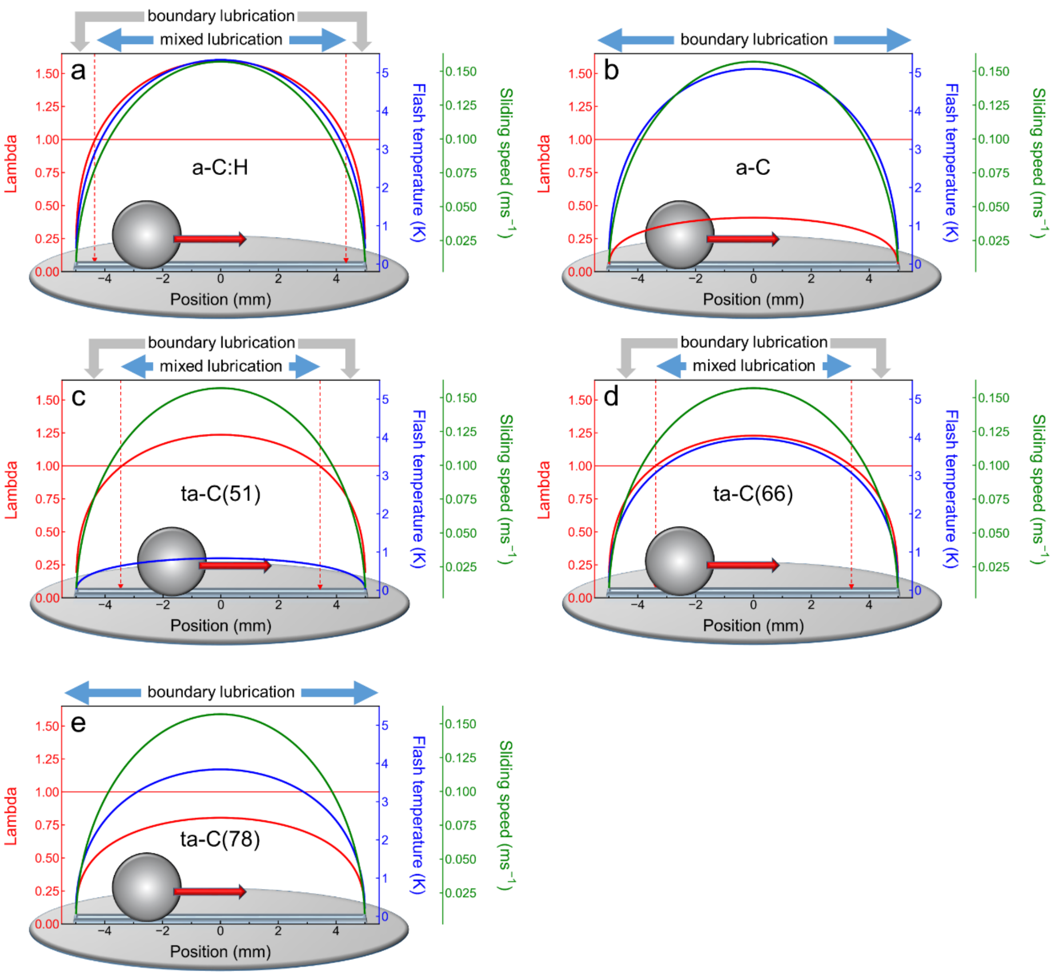


**Supplementary Fig. 9: Tallian's lambda (red), sliding speed (green), and flash temperature (blue) as a function of the cylinder's position over the stroke length ($-0.005 \leq x \leq 0.005$ m).** (a) a-C:H, (b) a-C, (c) ta-C(51), (d) ta-C(66), and (e) ta-C(78).

## Supplementary Note 10. Flash temperature calculation

Supplementary Table 4 shows DLC's structural, mechanical, and thermal parameters and averaged maximum flash temperatures $T_{\mathrm{fmax,BL}}$ in boundary lubrication. For a-C:H, $T_{\mathrm{fmax}}$ is about 5.3 °C at the center of the stroke, and decreases as the cylinder approaches to the ends of the stroke. The averaged maximum flash temperature in BL $T_{\mathrm{fmax,BL}}$ is about 2.3 °C. $T_{\mathrm{fmax,BL}}$ is the smallest for ta-C(51) due to ultralow friction ($\mu \approx 0.02$) and largest for a-C since BL applies over the entire stroke. This flash temperature rise is negligibly small for all DLC coatings, and plays little role in ZDDP's chemical reactions and observed tribological phenomena.

**Supplementary Table 4: Densities and thermal properties of all five DLC coatings and maximum flash temperature $T_{\mathrm{fmax,BL}}$ (°C) in boundary lubrication.**

| Label | Young's modulus $E$ (GPa) | Density $\rho$ (g cm$^{-3}$) | $sp^3$ C $p_{sp^3}$ (%) | Thermal conductivity $K$ (W m$^{-1}$ K$^{-1}$) | Heat capacity $C_p$ (J g$^{-1}$ K$^{-1}$) | Thermal diffusivity $D$ (10$^{-6}$ m$^2$ s$^{-1}$) | Maximum flash temperature in BL $T_{\mathrm{fmax,BL}}$ (°C) |
|---|---|---|---|---|---|---|---|
| a-C:H | 259 | 2.07 | - | 1.01 | 0.74 | 0.66 | 2.3 |
| a-C | 287 | 2.34 | 31 | 1.12 | 0.74 | 0.65 | 4.1 |
| ta-C(51) | 493 | 2.77 | 62 | 1.92 | 0.69 | 1.01 | 0.5 |
| ta-C(66) | 572 | 2.91 | 73 | 2.22 | 0.67 | 1.15 | 2.2 |
| ta-C(78) | 625 | 3.01 | 79 | 2.43 | 0.66 | 1.23 | 3.1 |

**Supplementary Note 11. Validation of the accuracy of the third-order density-functional tight-binding (DFTB3) quantum chemical force field**

DFTB3[4] is a semi-empirical quantum chemical method, and interatomic Slater-Koster parameters are fitted to ab-initio density-functional theory (DFT) calculations for a large set of molecules and crystals in terms of geometries, energies, forces and vibrational frequencies. Thus, it describes accurately bonding states in complicated chemical environments. For example, we have successfully employed DFTB for modeling of tribochemistry of organic friction modifiers and water with diamond[5] and diamond-like carbon surfaces[6]. However, it is important to validate the accuracy for each specific system.

We here performed ab-initio DFT calculations of a-C:H interacting with ZDDP. Quasi-static reference DFT calculations were performed using the CP2K code[7], where the mixed Gaussian Plane Wave (GPW) method was employed[8]. A plane wave cutoff of 500 Ry was chosen to define the grid spacing. All calculations were performed within the Perdew-Burke-Enzerhof (PBE) approximation[9] to the exact exchange correlation functional. Gaussian double-zeta basis sets with polarization functions were used to expand the Kohn-Sham wave functions of the valence electrons

and Goedecker-Teter-Hutter pseudopotentials[10] were applied to effectively treat the core electrons. Periodic boundary conditions were imposed along all spatial directions.

In order to obtain statistics about the formation of C−S bonds and the splitting-off of single S atoms from the ZDDP-molecule, 25 different initial configurations were generated by translating the initial position of the ZDDP-molecule on a regular lateral 5×5 grid. Normal pressure in the simulation box was successively increased by decreasing the simulation box size in the direction perpendicular to the a-C:H slab in steps of 0.5 Å. After each step the atom positions were relaxed until the convergence criterion of a maximal atomic force of 0.01 eV $Å^{-1}$ was reached.

Supplementary Figure 10 shows averaged numbers of S−C bonds $\langle \Delta n_{\mathrm{S-C}} \rangle$ formed between ZDDP sulphur and DLC carbon atoms and sulphur atoms released from ZDDP $\langle n_{\mathrm{Sulphur}} \rangle$ to the DLC as a function of $P_z$ obtained from both DFTB3 and ab-initio DFT PBE calculations. Although DFTB3 slightly overestimates these numbers, the results are in good agreement between DFTB3 and DFT PBE calculations in terms of a linear increase in both quantities on the contact pressure $P_z$. It thus confirms that DFTB3 is able to describe mechanochemical decomposition of ZDDP on DLC surfaces accurately.

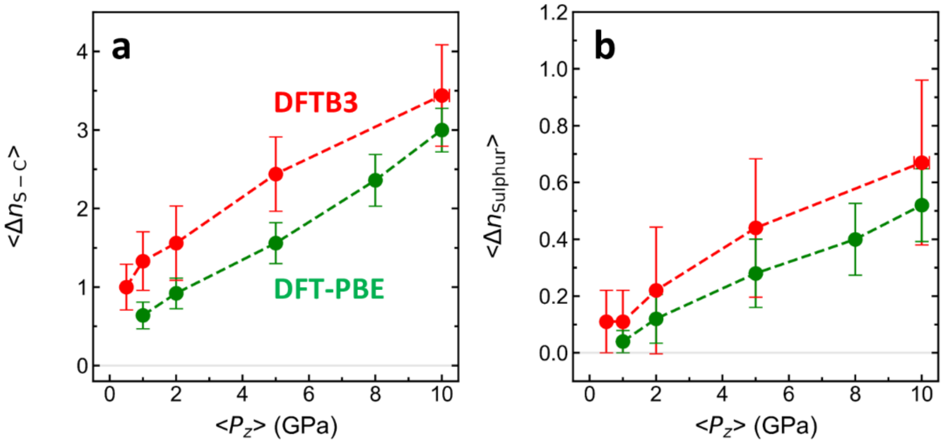


**Supplementary Fig. 10: Comparison of DFTB3 with ab-initio DFT-PBE calculations for quasi-static contact-closing of unpassivated a-C:H surfaces interacting with a ZDDP molecule.** Averaged numbers of (a) S−C bonds $\langle \Delta n_{\mathrm{S-C}} \rangle$ formed between ZDDP sulphur and DLC carbon atoms as well as (b) sulphur atoms $\langle n_{\mathrm{Sulphur}} \rangle$ released from ZDDP to the DLC after opening the contact as a function of the contact pressure $P_z$ for DFTB3 (red) and DFT-PBE

(green). For both DFTB3 and DFT-PBE, the same a-C:H samples were employed as in Fig. 6. The error bars represent standard error of the means.

**Supplementary References**


1. Zhang, J., Ewen, J. P., Ueda, M., Wong, J. S. S. & Spikes, H. A. Mechanochemistry of Zinc Dialkyldithiophosphate on Steel Surfaces under Elastohydrodynamic Lubrication Conditions. *ACS Appl. Mater. Interfaces* **12**, 6662–6676 (2020).
2. Persson, B. N. J. Theory of rubber friction and contact mechanics. *J. Chem. Phys.* **115**, 3840–3861 (2001).
3. Jacobs, T. D. B., Junge, T. & Pastewka, L. Quantitative characterization of surface topography using spectral analysis. *Surf. Topogr. Metrol. Prop.* **5**, 013001 (2017).
4. Gaus, M., Cui, Q. & Elstner, M. DFTB3: Extension of the Self-Consistent-Charge Density-Functional Tight-Binding Method (SCC-DFTB). *J. Chem. Theory Comput.* **7**, 931–948 (2011).
5. Kuwahara, T., Moras, G. & Moseler, M. Friction Regimes of Water-Lubricated Diamond (111): Role of Interfacial Ether Groups and Tribo-Induced Aromatic Surface Reconstructions. *Phys. Rev. Lett.* **119**, 096101 (2017).
6. Kuwahara, T. *et al.* Mechano-chemical decomposition of organic friction modifiers with multiple reactive centres induces superlubricity of ta-C. *Nat. Commun.* **10**, 151 (2019).
7. VandeVondele, J. *et al.* Quickstep: Fast and accurate density functional calculations using a mixed Gaussian and plane waves approach. *Comput. Phys. Commun.* **167**, 103–128 (2005).
8. Lippert, G., Hutter, J. & Parrinello, M. A hybrid Gaussian and plane wave density functional scheme. *Mol. Phys.* **92**, 477–487 (1997).
9. Perdew, J. P., Burke, K. & Ernzerhof, M. Generalized Gradient Approximation Made Simple. *Phys. Rev. Lett.* **77**, 3865–3868 (1996).
10. Goedecker, S., Teter, M. & Hutter, J. Separable dual-space Gaussian pseudopotentials. *Phys. Rev. B* **54**, 1703–1710 (1996).
11. Kunze, T. *et al.* Wear, plasticity, and rehybridization in tetrahedral amorphous carbon. *Tribol. Lett.* **53**, 119–126 (2014).
12. Moes, H. Optimum similarity analysis with applications to elastohydrodynamic lubrication. *Wear* **159**, 57–66 (1992).
13. Tallian, T. E. On Competing Failure Modes in Rolling Contact. *ASLE Trans.* **10**, 418–439 (1967).
