# Supplementary material for: Interplay of mechanics and chemistry governs wear of diamond-like carbon coatings interacting with ZDDP-additivated lubricants
Source: Nat Commun. 2021 Jul 27;12:4550. doi: 10.1038/s41467-021-24766-6 (PMC8316475; doi:10.1038/s41467-021-24766-6)
Supplement: Supplementary file 3 — Description of Additional Supplementary Files [file 41467_2021_24766_MOESM3_ESM.pdf]

Description of Additional Supplementary Files

File Name: Supplementary Movie 1
Description: Quantum molecular dynamics trajectory of a-C:H surfaces interacting with a ZDDP molecule.

File Name: Supplementary Movie 2
Description: Quantum molecular dynamics trajectory of a-C surfaces interacting with a ZDDP molecule.
